# Triglyceride-glucose index as a marker for visceral obesity in patients with gastric cancer

**DOI:** 10.3389/fnut.2024.1515918

**Published:** 2025-01-10

**Authors:** Junbo Zuo, Zhenhua Huang, Yan Ge, Xin Ding, Xiuhua Wang, Yan Huang

**Affiliations:** ^1^Department of General Surgery, The Affiliated People’s Hospital of Jiangsu University, Zhenjiang, China; ^2^Department of Nutrition, The Affiliated People’s Hospital of Jiangsu University, Zhenjiang, China; ^3^Department of Cardiovascular Medicine, The Affiliated People’s Hospital of Jiangsu University, Zhenjiang, China

**Keywords:** triglyceride–glucose index, insulin resistance, visceral fat, visceral obesity, gastric cancer

## Abstract

**Background:**

The triglyceride-glucose (TyG) index has emerged as a validated and cost-effective indicator of insulin resistance (IR). Given the significant association between visceral obesity and IR, this study aimed to investigate the utility of the TyG index in estimating visceral obesity in patients with gastric cancer (GC).

**Methods:**

The visceral fat area (VFA), subcutaneous fat area (SFA), and VFA-to-SFA ratio (VSR) were determined through the analysis of CT images at the lumbar 3 level. The definition of visceral obesity was established as VFA ≥ 100 cm^2^. The association between the TyG index and visceral obesity was assessed using logistic regression analysis and restricted cubic splines. The diagnostic performance for identifying visceral obesity was evaluated by calculating the area under the Receiver Operating Characteristics curve (AUC).

**Results:**

The cross-sectional study enrolled a total of 314 patients with GC, among whom 159 (50.64%) were identified as having visceral obesity. The TyG index was positively correlated with VFA (*r* = 0.45, *p* < 0.001), SFA (*r* = 0.23, *p* < 0.001), and VSR (*r* = 0.35, *p* < 0.001). However, subsequent multivariate linear regression analysis demonstrated that the TyG index was significantly associated with VFA and VSR, but not SFA. After adjusting for potential confounding factors, the TyG index remained independently associated with visceral obesity (OR = 2.54, 95% CI: 1.32–4.89, *p* = 0.005) and demonstrated a significantly positive linear correlation with visceral obesity in patients with GC (*p*-value for non-linearity = 0.116). TyG-BMI, the combination index of TyG and BMI, showed the highest predictive power in identifying visceral obesity in GC patients (AUC = 0.849, 95% CI: 0.807–0.890, *p* < 0.001). The subgroup analysis revealed a significantly stronger positive association between the TyG index and visceral obesity in patients with BMI ≥ 25 kg/m^2^ (*p* for interaction = 0.049).

**Conclusion:**

The TyG index exhibited a significant association with visceral obesity and proved to be a valuable predictor for visceral obesity when combined with BMI in patients with GC.

## Introduction

Gastric cancer (GC) ranks fifth worldwide in terms of both incidence and mortality, with over 968,000 new cases and nearly 660,000 deaths in 2022 (1). The radical gastrectomy, which involves the resection of both the primary tumor and regional lymph nodes, remains the fundamental and most effective treatment for GC; however, it is associated with potential postoperative complications that can significantly impact patients’ quality of life, tolerance to chemotherapy, and overall survival (2). Moreover, even after curative resection surgery, advanced-stage GC patients still face the persistent risk of recurrence and metastasis. Therefore, accurate prediction of complication risks and tumor prognosis plays a pivotal role in formulating appropriate treatment strategies.

Obesity is associated with metabolic syndrome, type 2 diabetes and visceral fat, which can significantly influence surgical outcomes following radical gastrectomy (3). Although body mass index (BMI) is widely used as an anthropometric index of obesity, it fails to accurately reflect the distribution of fat tissue within the body. Compared with BMI, visceral fat has been reported to be more optimal for the evaluation of surgical outcomes (4). Furthermore, it has been demonstrated that visceral obesity is associated with an increased incidence of postoperative complications and a poorer prognosis among patients undergoing radical gastrectomy (5). Additionally, metabolic syndrome and visceral fat are the first to improve after metabolic bariatric surgery, making visceral fat a modifiable factor (6). Thus, timely screening and assessment of visceral obesity is crucial prior to surgical treatment in clinical practice.

Computed tomography (CT) is considered the gold standard for non-invasive assessment of body composition, including subcutaneous and visceral fat mass (7). Patients with GC are required to undergo routine CT scans for tumor staging assessment before surgery and for postoperative follow-up. Consequently, assessing visceral obesity in GC patients using CT is feasible; however, it should be noted that this approach is expensive and time-consuming, requires specialized software and highly skilled personnel, and most importantly exposes patients to radiation (8, 9). Therefore, implementing this method as a routine practice or for dynamic surveillance in the clinical setting is not feasible.

Recently, the triglyceride-glucose (TyG) index, derived from the calculation of triglyceride (TG) and fasting blood glucose (FBG), has emerged as a surrogate biochemical indicator for the assessment of insulin resistance (IR) (10, 11). The association between obesity and the development of IR has been firmly established (12). Furthermore, previous studies have indicated that visceral obesity plays a pivotal role in determining IR (13). Given the significant relationship between visceral obesity and insulin resistance, the TyG index may serve as a valuable biomarker for identifying visceral obesity in clinical settings. However, limited research has been conducted to evaluate the association between the TyG index and visceral obesity in the disease states. Therefore, this cross-sectional study aimed to investigate the utility of the TyG index in estimating visceral obesity in patients with GC.

## Methods

### Study patients

As described in our previous study (14), this cross-sectional study included consecutive patients diagnosed with GC in our department between October 2021 and March 2023. The inclusion criteria are as follows: (1) histological confirmation of gastric adenocarcinoma by endoscopic biopsy, (2) age ranging from 18 to 80 years, and (3) no neoadjuvant therapy received prior to admission. The exclusion criteria were as follows: (1) missing abdominal CT scans from our institution; (2) absence of major laboratory data, such as TG and FBG; (3) patients with severe comorbidities including chronic kidney disease, heart failure, or liver cirrhosis; and (4) concurrent occurrence or history of other malignancies within the past 5 years.

### Data collection

The demographic and clinical data, including sex, age, body mass index (BMI), comorbidities (hypertension and diabetes), performance status, and pathological staging were extracted from electronic medical records. Performance status was assessed based on the Eastern Cooperative Oncology Group (ECOG) grade. Pathological staging followed the Eighth edition of the American Joint Committee on Cancer (AJCC) tumor-node-metastasis (TNM) staging system. The blood routine and biochemical tests, including C-reactive protein (CRP), neutrophil and lymphocyte counts, hemoglobin levels, fasting plasma glucose (FPG), high-density lipoproteins (HDL), low-density lipoproteins (LDL), total cholesterol (TC), triglyceride (TG) levels, and serum albumin levels were conducted within 48 h of admission. The neutrophil/lymphocyte ratio (NLR) was calculated by dividing the neutrophil count by the lymphocyte count. Anemia was defined as a hemoglobin level < 120 g/L for males and < 110 g/L for females, while hypoproteinemia was defined as a serum albumin level < 35 g/L. The TyG index was calculated using the formula Ln [TG (mg/dL) × FPG (mg/dL) ÷ 2] (15). Subsequently, the TyG-BMI index was derived by multiplying the TyG index with BMI.

### CT-based measurement of visceral and subcutaneous fat

As shown in Supplementary Figure S1, the Slice-O-Matic software version 5.0 (Tomovision, Magog, QC, Canada) was utilized for the analysis of CT images at the level of lumbar 3 (L3) to obtain measurements for visceral fat area (VFA, cm^2^) and subcutaneous fat area (SFA, cm^2^). Tissue-specific Hounsfield units (HU) thresholds ranging from −190 to −30 HU were employed for subcutaneous fat, while thresholds of −150 to −50 HU were utilized for visceral fat. The VFA-to-SFA ratio (VSR) was determined by calculating the quotient of VFA and SFA. Visceral obesity was defined as VFA ≥ 100 cm^2^, according to the criteria established by the Japan Society for the Study of Obesity, which is widely acknowledged in clinical settings (16).

### Statistical analysis

Continuous variables with a normal distribution (determined by the Kolmogorov–Smirnov test) were represented as the mean ± standard deviation (SD), while those with a non-normal distribution were reported as the median and interquartile range (IQR). Categorical variables were presented in terms of number and percentage. According to the tertiles of the TyG index, the patients were categorized into three groups: low TyG (< 8.25), middle TyG (> 8.25, ≤ 8.70), and high TyG (> 8.70). The comparison among these three groups was conducted using one-way ANOVA for normally distributed continuous variables, Kruskal-Wallis tests for non-normally distributed continuous variables, and chi-square tests for categorical variables. The Pearson correlation analysis was utilized to examine the correlation between the TyG index and clinical characteristics, and linear regression analyses were performed to assess the relationship between the TyG index and VFA, SFA, and VSR. Univariable and multivariable logistic regression analyses were further conducted to assess the association between the TyG index and visceral obesity. The variables that exhibited a significance level of *p* < 0.10 in the univariable analysis and showed no indications of multicollinearity (Variance Inflation Factor < 10) were included in the multivariable analysis. Additionally, restricted cubic splines (RCS) were employed to investigate the dose–response relationship (linear or nonlinear) between the TyG index and visceral obesity. The diagnostic performance for identifying visceral obesity was evaluated by constructing Receiver Operating Characteristics (ROC) analysis and calculating the area under the ROC curve (AUC). The optimal cut-off value was determined using the formula of maximal Youden’s index. Statistical differences among the AUCs were compared using the DeLong test (17). The subgroup analyses were performed based on sex, age (<65 years or ≥ 65 years), BMI (< 25 kg/m^2^ or ≥ 25 kg/m^2^), hypertension, and diabetes. The likelihood ratio test was utilized to assess the interaction among these subgroups. The statistical analysis was conducted using SPSS version 25.0 (IBM Corp, Armonk, NY, USA) and MedCalc 20.03. A two-tailed *p*-value of less than 0.05 was considered statistically significant.

## Results

### Demographic and clinical characteristics

**Figure 1:**
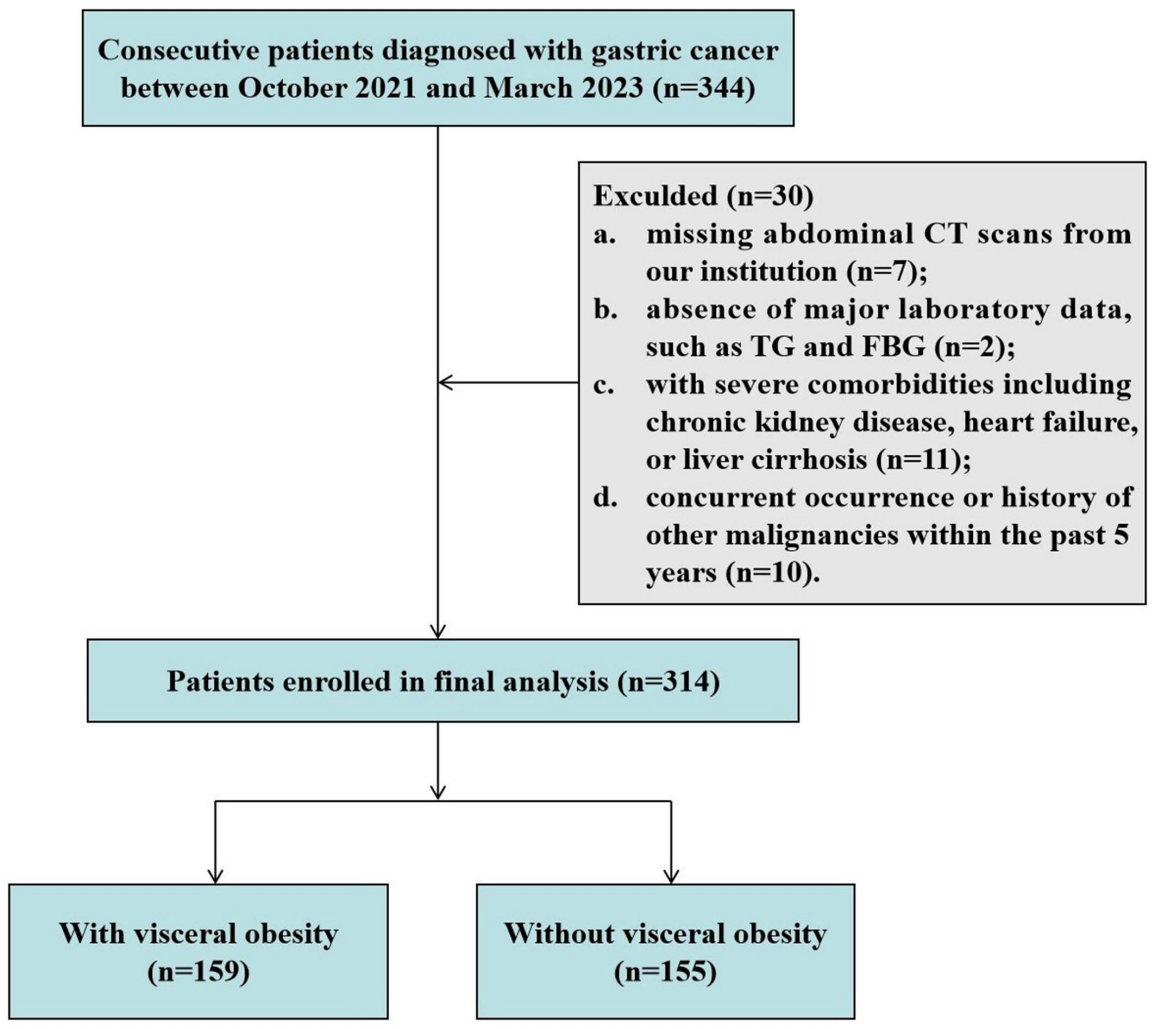
Flowchart of study subject enrollment. CT, computed tomography; FPG, fasting plasma glucose; TG, triglyceride.

As shown in Figure 1, a total of 314 patients diagnosed with GC were enrolled in this study. Among them, there were 92 (29.3%) female patients and 222 (70.7%) male patients, with a median age of 68 years (IQR: 62–72) and an average BMI of 23.43 ± 3.34 kg/m^2^. There was a clear sex disparity in the distribution of adipose tissue, with male patients exhibiting significantly higher levels of VFA [119.20 (66.18–188.66) cm^2^ vs. 92.01 (65.07–121.89) cm^2^, *p* = 0.005] and VSR [1.17 (0.85–1.73) vs. 0.63 (0.48–0.81), *p* < 0.001], while demonstrating lower levels of SFA [94.87 (62.27–125.51) cm^2^ vs. 142.83 (103.99–182.44) cm^2^, *p* < 0.001] compared to female patients (Supplementary Figure S2A).

The demographic and clinical characteristics of patients with GC are presented in Table 1. Based on the tertiles of the TyG index, the patients were classified into three groups: low TyG (<8.25, *n* = 105), middle TyG (>8.25, ≤8.70, *n* = 105), and high TyG (>8.70, *n* = 104). Patients with higher baseline TyG index exhibited an increased prevalence of hypertension, diabetes, favorable ECOG performance status, early TNM stage, use of hypoglycemic drugs, and lipid-lowering drugs (all *p* < 0.05). Furthermore, they demonstrated reduced levels of HDL and elevated levels of BMI, TC, and LDL (all *p* < 0.05). Moreover, the levels of VFA, SFA, and VSR significantly increased from the low to high tertiles of the TyG index (all *p* < 0.001; Supplementary Figure S2B and Table 1).

**Table 1:** Clinical characteristics of the GC patients based on TyG tertiles.

Variables	Total	TyG index level			*p*-value
		Low (≤8.25)	Middle (>8.25, ≤8.70)	High (>8.70)	
*n*	314	105	105	104	
Sex, *n* (%)					0.476
Female	92 (29.30)	27 (25.71)	35 (33.33)	30 (28.85)	
Male	222 (70.70)	78 (74.29)	70 (66.67)	74 (71.15)	
Age, years	68 (62, 72)	68 (64, 72)	68 (64, 73)	66 (59, 72)	0.270
BMI, kg/m^2^	23.43 ± 3.34	22.18 ± 3.12	23.66 ± 3.36	24.47 ± 3.15	<0.001
Hypertension, *n* (%)	150 (47.77)	42 (40.00)	48 (45.71)	60 (57.69)	0.033
Diabetes, *n* (%)	43 (13.69)	6 (5.71)	9 (8.57)	28 (26.92)	<0.001
ECOG performance status, *n* (%)					0.016
0	189 (60.19)	53 (50.48)	66 (62.86)	70 (67.31)	
1	83 (26.43)	29 (27.62)	27 (25.71)	27 (25.96)	
≥2	42 (13.38)	23 (21.90)	12 (11.43)	7 (6.73)	
TNM stage, *n* (%)					0.023
I	77 (24.52)	16 (15.24)	23 (21.90)	38 (36.54)	
II	61 (19.43)	22 (20.95)	21 (20.00)	18 (17.31)	
III	134 (42.68)	51 (48.57)	44 (41.90)	39 (37.50)	
IV	42 (13.38)	16 (15.24)	17 (16.19)	9 (8.65)	
Hypoproteinemia, *n* (%)	96 (30.57)	38 (36.19)	34 (32.38)	24 (23.08)	0.107
Anemia, *n* (%)	134 (42.68)	53 (50.48)	44 (41.90)	37 (35.58)	0.092
CRP ≥ 5 mg/L, *n* (%)	67 (21.34)	19 (18.10)	26 (24.76)	22 (21.15)	0.498
NLR	2.37 (1.81, 3.32)	2.29 (1.82, 3.28)	2.38 (1.91, 3.22)	2.38 (1.72, 3.33)	0.921
FPG, mmol/L	5.19 (4.75, 5.94)	4.84 (4.45, 5.19)	5.15 (4.81, 5.62)	5.98 (5.31, 6.95)	<0.001
TG, mmol/L	1.12 (0.84, 1.57)	0.77 (0.62, 0.87)	1.18 (1.02, 1.32)	1.79 (1.54, 2.13)	<0.001
TC, mmol/L	4.11 ± 0.92	3.75 ± 0.78	4.11 ± 0.89	4.48 ± 0.93	<0.001
HDL, mmol/L	1.17 (0.99, 1.41)	1.27 (1.08, 1.51)	1.21 (0.99, 1.39)	1.06 (0.91, 1.20)	<0.001
LDL, mmol/L	2.21 (1.82, 2.69)	1.93 (1.65, 2.25)	2.21 (1.89, 2.69)	2.58 (2.18, 2.96)	<0.001
TyG index	8.51 ± 0.55	7.93 ± 0.27	8.48 ± 0.13	9.12 ± 0.32	<0.001
TyG-BMI index	199.89 ± 34.61	175.97 ± 26.36	200.68 ± 28.92	223.24 ± 30.97	<0.001
VFA, cm^2^	102.25 (65.91, 165.41)	75.94 (32.23, 108.15)	108.35 (63.34, 162.10)	147.38 (92.05, 212.38)	<0.001
SFA, cm^2^	106.07 (71.71, 142.25)	89.64 (56.50, 122.55)	108.45 (60.76, 147.25)	112.33 (89.70, 149.55)	<0.001
VSR	0.98 (0.65, 1.47)	0.81 (0.56, 1.13)	1.02 (0.63, 1.38)	1.16 (0.82, 1.86)	<0.001
Visceral obesity, *n* (%)	159 (50.64)	30 (28.57)	57 (54.29)	72 (69.23)	<0.001
Use of hypoglycemic drugs, *n* (%)	36 (11.46)	6 (5.71)	9 (8.57)	21 (20.19)	0.002
Use of lipid-lowering drugs, *n* (%)	9 (2.87)	0 (0)	2 (1.90)	7 (6.73)	0.006

BMI, body mass index; CRP, C-reactive protein; ECOG, Eastern cooperative oncology group; FPG, fasting plasma glucose; HDL, high-density lipoproteins; LDL, low-density lipoproteins; NLR, neutrophil-to-lymphocyte ratio; SFA, subcutaneous fat area; TC, total cholesterol; TG, triglyceride; TNM, tumor–node–metastasis; TyG, triglyceride and glucose index; VFA, visceral fat area; VSR, VFA-to-SFA ratio.

### Correlations between TyG index and clinical characteristics

**Figure 2:**
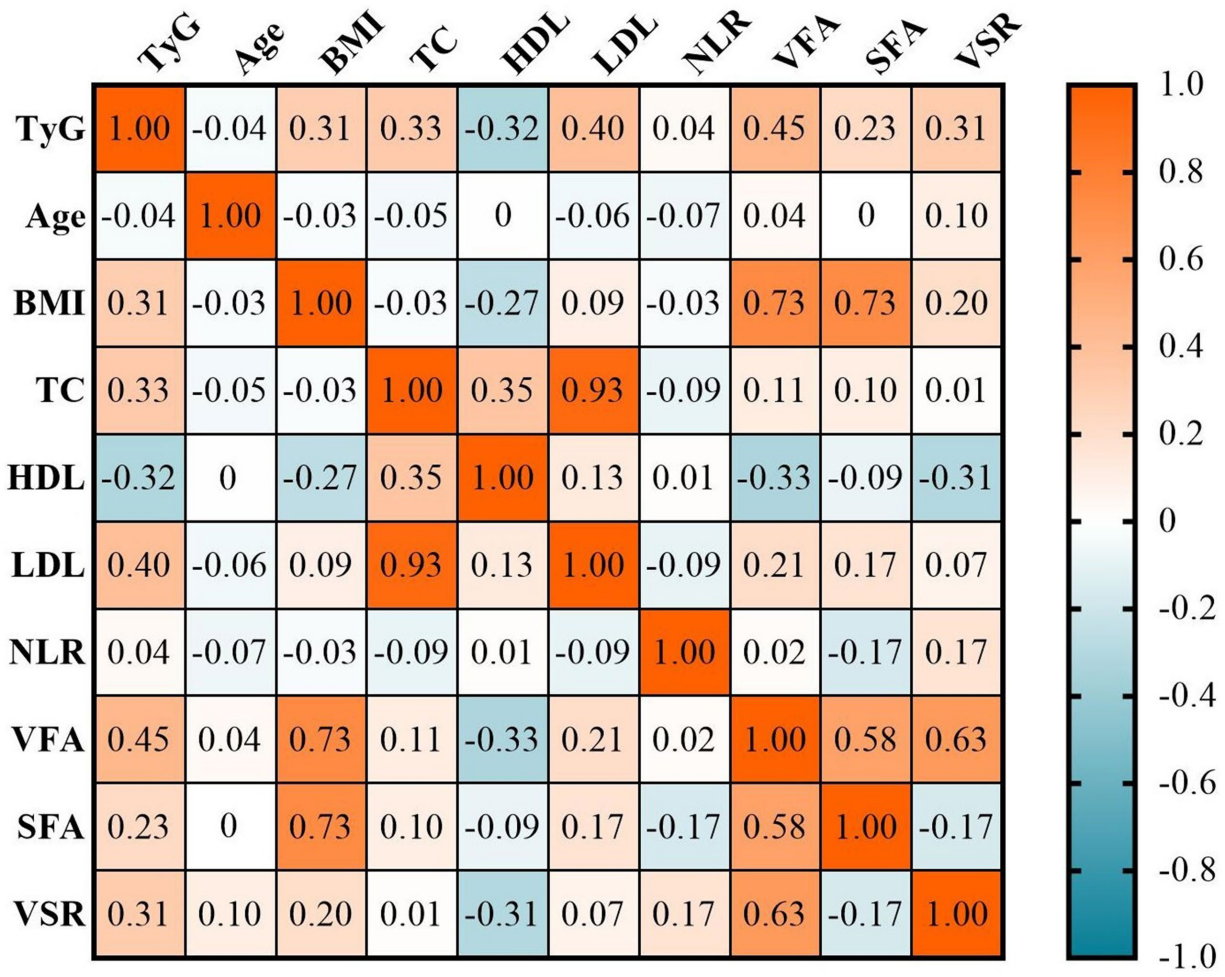
The correlations between the TyG index and clinical characteristics. BMI, body mass index; HDL, high-density lipoproteins; LDL, low-density lipoproteins; NLR, neutrophil-to-lymphocyte ratio; SFA, subcutaneous fat area; TC, total cholesterol; TyG, triglyceride and glucose index; VFA, visceral fat area; VSR, VFA-to-SFA ratio.

The correlations between the TyG index and clinical characteristics were illustrated in Figure 2. The TyG index exhibited significantly positive correlations with BMI (*r* = 0.31, *p* < 0.001), total cholesterol (*r* = 0.33, *p* < 0.001), LDL (*r* = 0.40, *p* < 0.001), and negative correlation with HDL (*r* = −0.32, *p* < 0.001). Additionally, there were also positive correlations observed between the TyG index and adipose tissue measures, including VFA (*r* = 0.45, *p* < 0.001), SFA (*r* = 0.23, *p* < 0.001), and VSR (*r* = 0.35, *p* < 0.001). Notably, the correlation between the TyG index and VSR was stronger compared to that of BMI (*r* = 0.31 vs. *r* = 0.20). Furthermore, in multivariate linear regression analysis adjusted for age, sex, BMI, hypertension, diabetes, ECOG performance status, TNM stage, hypoproteinemia, and anemia, a significant correlation was found between the TyG index and VFA and VSR (both *p* < 0.05), but not SFA (*p* > 0.05; Table 2).

**Table 2:** Univariable and multivariable linear regression analysis of TyG index (independent variable) and VFA, SFA, and VSR (dependent variable).

Variables	Model I		Model II		Model III	
	*β* (95% CI)	*p*-value	*β* (95% CI)	*p*-value	*β* (95% CI)	*p*-value
VFA, cm^2^	57.32 (43.70–70.94)	<0.001	31.09 (21.24–40.95)	<0.001	29.35 (18.81–39.89)	<0.001
SFA, cm^2^	23.84 (12.14–35.54)	<0.001	23.84 (12.14–35.54)	0.885	−2.71 (−10.05–4.63)	0.470
VSR	0.35 (0.22–0.47)	<0.001	0.33 (0.21–0.44)	<0.001	0.32 (0.19–0.44)	<0.001

Model I: non-adjusted.

Model II: adjusted for age, sex, and BMI.

Model III: adjusted for age, sex, BMI, hypertension, diabetes mellitus, ECOG performance status, TNM stage, hypoproteinemia and anemia.

BMI, body mass index; ECOG, Eastern cooperative oncology group; SFA, subcutaneous fat area; TNM, tumor–node–metastasis; TyG, triglyceride and glucose index; VFA, visceral fat area; VSR, VFA-to-SFA ratio.

### Association between TyG index and visceral obesity

**Figure 3:**
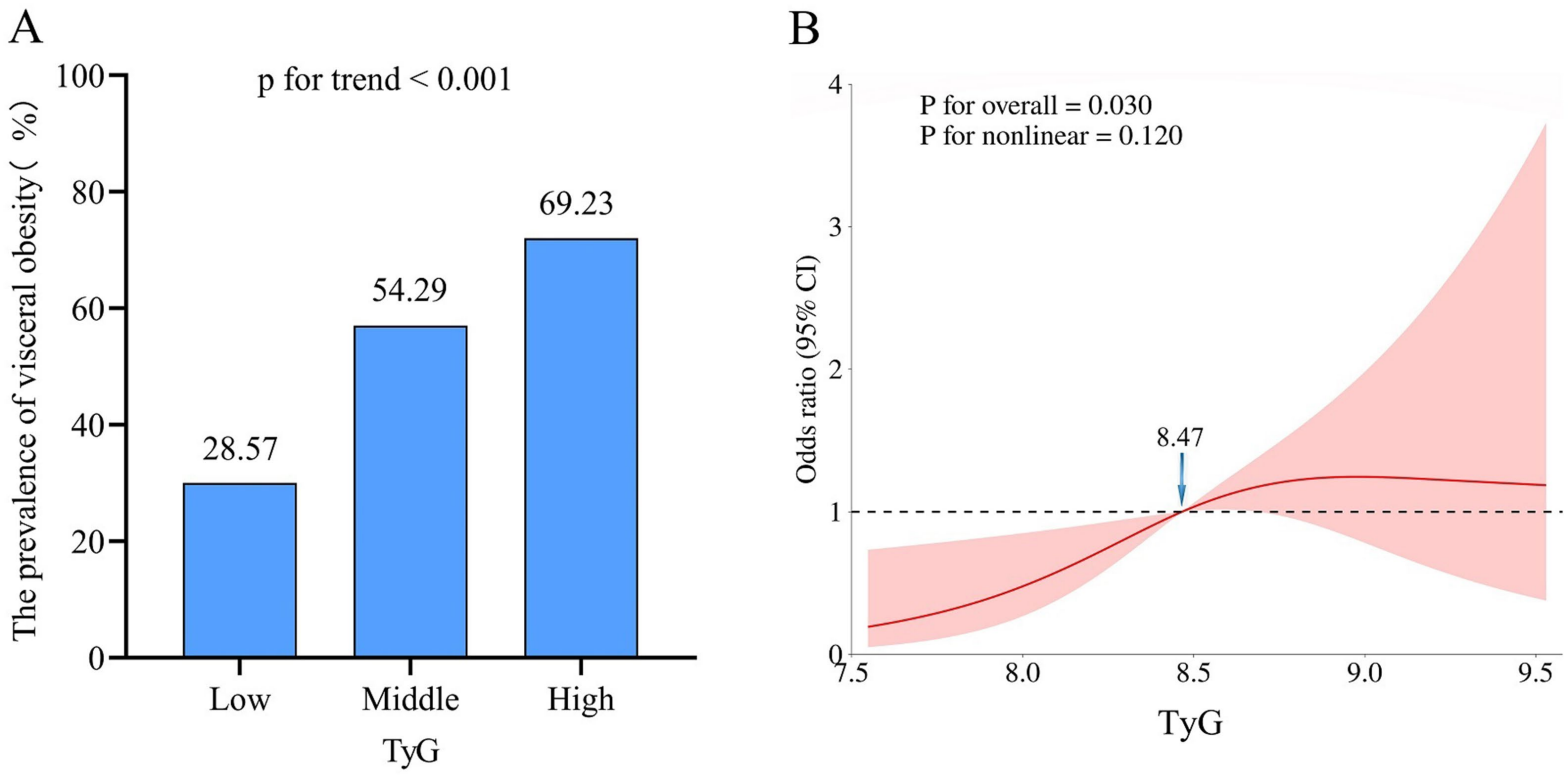
Association between TyG index and visceral obesity. **(A)** Prevalence of visceral obesity according to tertiles of the TyG index. **(B)** Restricted cubic spline plot between the TyG index and visceral obesity. TyG, triglyceride and glucose index.

**Figure 4:**
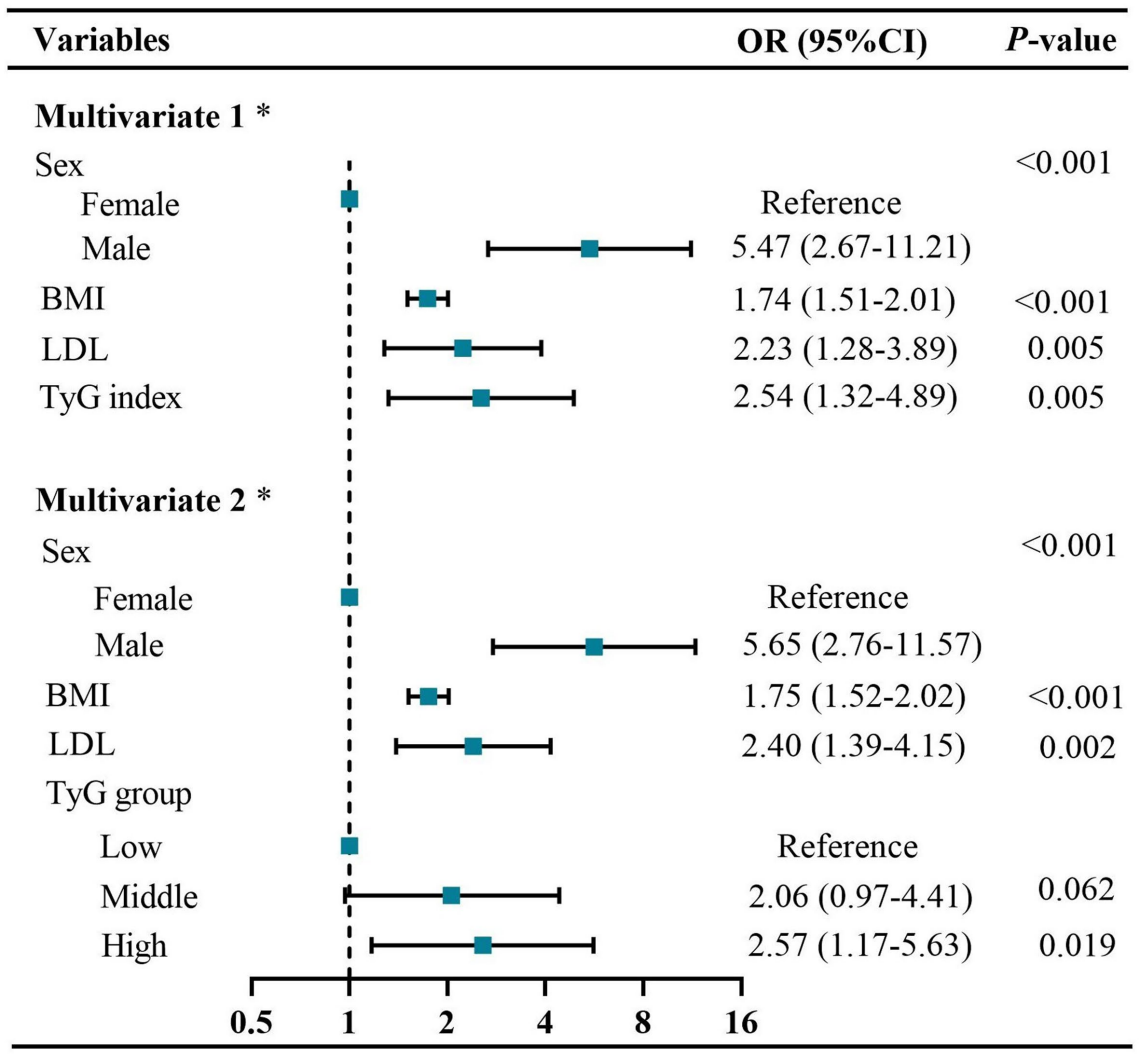
Forest plots of independent factors associated with visceral obesity in GC patients. Multivariate 1: using TyG index as a continuous variable. Multivariate 2: using TyG index as a categorical variable. * Adjusted for sex, BMI, hypertension, diabetes, CRP ≥ 5 mg/L, ECOG performance status, TNM stage, anemia, TC, HDL, and LDL. BMI, body mass index; CRP, C-reactive protein; CI, confidence interval; ECOG, Eastern cooperative oncology group; HDL, high-density lipoproteins; LDL, low-density lipoproteins; OR, odds ratio; TC, total cholesterol; TNM, tumor–node–metastasis; TyG, triglyceride and glucose index.

The prevalence of visceral obesity was observed in 50.6% of all patients, with 28.57% in the low-TyG group, 54.29% in the middle-TyG group, and 69.23% in the high-TyG group, respectively (*p* < 0.001; Figure 3A and Table 1). The univariate logistic regression analysis showed that sex, BMI, hypertension, ECOG performance status, TNM stage, anemia, total cholesterol, HDL, LDL and TyG index (as both continuous and categorical variables) were statistically associated with visceral obesity (all *p* < 0.05; Supplementary Table S1). After adjusting for confounding factors, the TyG index as a continuous variable remained independently associated with visceral obesity (OR = 2.54, 95% CI: 1.32–4.89, *p* = 0.005; Figure 4, multivariate 1). Furthermore, compared to the low-TyG group, the high-TyG group exhibited a 2.57-fold increased risk of visceral obesity (OR = 2.57, 95% CI: 1.17–5.63, *p* = 0.019) in the multivariate logistic regression analysis (Figure 4, multivariate 2).

**Figure 5:**
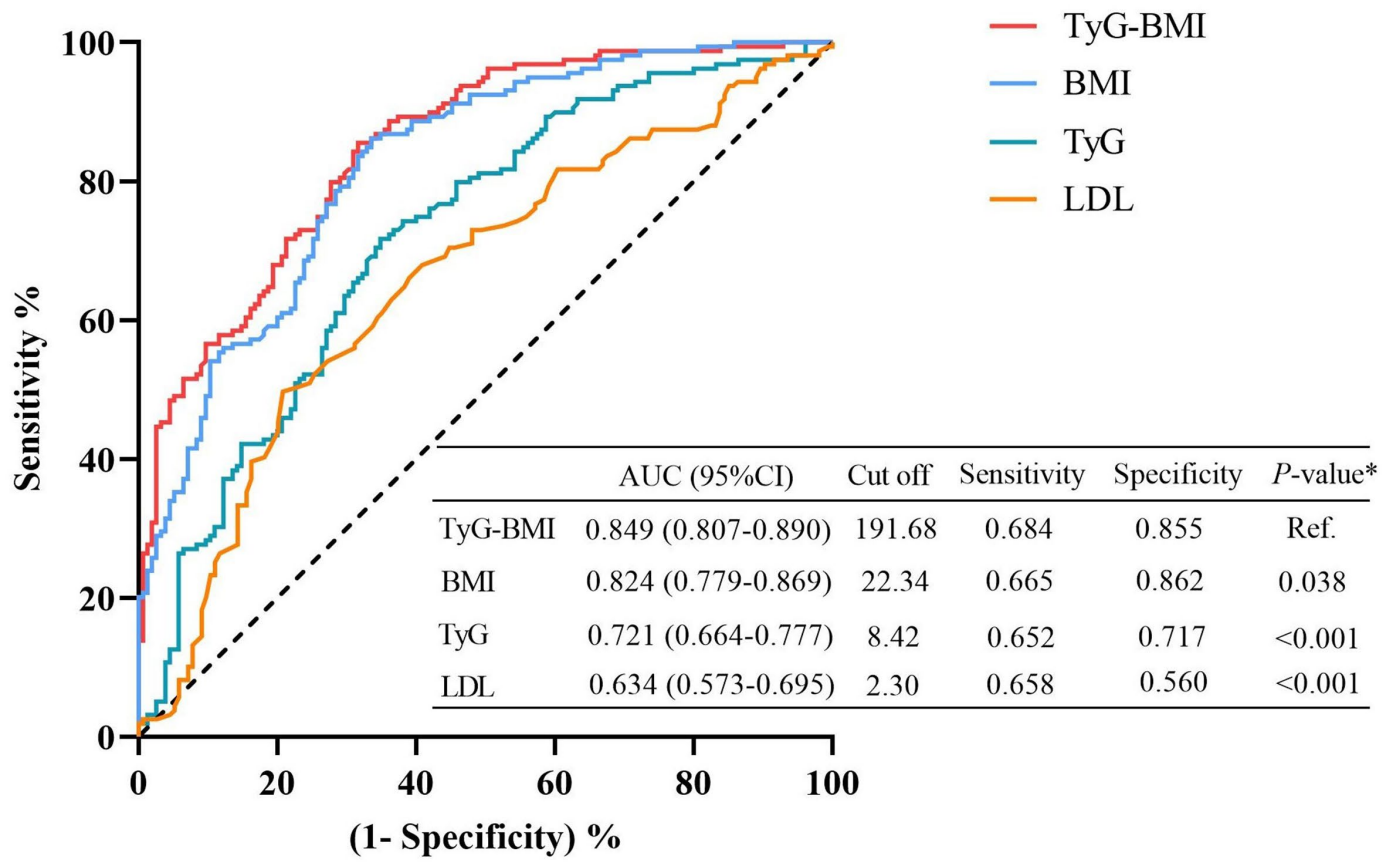
Receiver operating characteristic (ROC) analyses for visceral obesity. The predictive performances were calculated using Delong’s test and compared to TyG-BMI. BMI, body mass index; CI, confidence interval; LDL, low-density lipoproteins; TyG, triglyceride and glucose index.

The restricted cubic spline analysis revealed a significant positive linear association between the TyG index and visceral obesity after adjusting for potential confounding factors (p for non-linearity = 0.116; Figure 3B). Notably, an increased risk of developing visceral obesity was observed for TyG index values exceeding 8.47. As depicted in the ROC curve analysis shown in Figure 5, the combination index of TyG and BMI, known as TyG-BMI, exhibited the highest predictive power (AUC = 0.849, 95% CI: 0.807–0.890, *p* < 0.001) compared to BMI (AUC = 0.824, 95% CI: 0.779–0.869, *p* < 0.001), TyG (AUC = 0.721, 95% CI: 0.664–0.777, *p* < 0.001), and LDL levels (AUC = 0.634, 95% CI: 0.573–0.695, *p* < 0.001) in predicting visceral obesity in GC patients.

### Subgroup analyses

**Figure 6:**
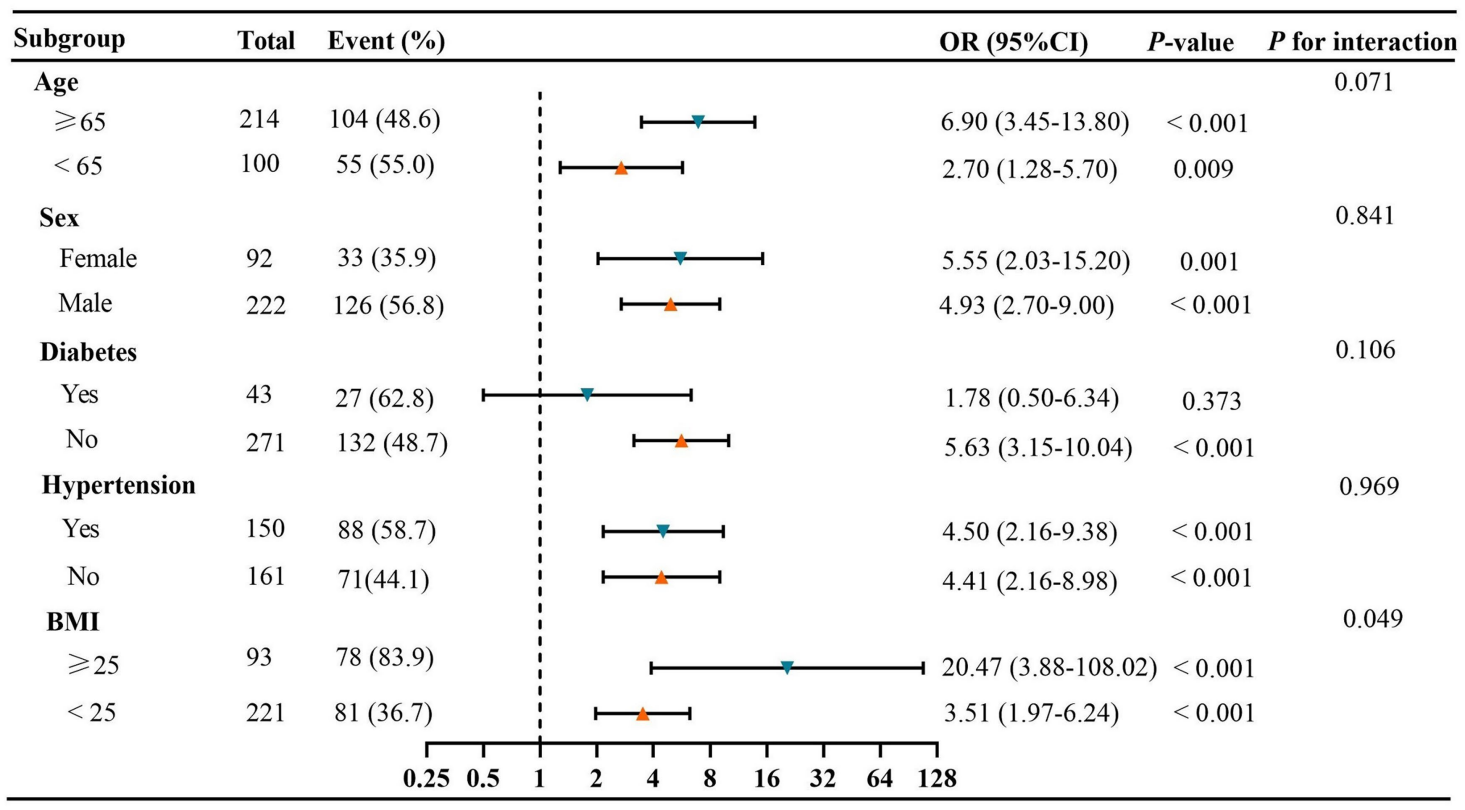
Subgroup and interaction analyses between the TyG index and visceral obesity. Event (%), the number and prevalence of Visceral obesity. BMI, body mass index; CI, confidence interval; OR, odds ratio; TyG, triglyceride-glucose.

The subgroup analysis revealed a significant positive association between the TyG index and the risk of visceral obesity, except in GC patients with diabetes (*p* > 0.05; Figure 6). Additionally, there were no significant interactions observed between the TyG index and visceral obesity when stratifying by age, sex, hypertension, or diabetes (All p for interaction >0.05; Figure 6). However, our results indicated that BMI could modify the association between the TyG index and visceral obesity (p for interaction = 0.049), with a stronger association observed in GC patients with BMI ≥ 25 kg/m^2^.

## Discussion

In this cross-sectional study, we investigated the association between the TyG index and visceral obesity in patients with GC. Our results revealed significant positive correlations between the TyG index and measures of adipose tissue, including VFA, SFA, and VSR. However, subsequent multivariate linear regression analysis demonstrated that the TyG index was significantly associated with VFA and VSR, but not SFA. Furthermore, our study established an independent association between the TyG index and visceral obesity, even after adjusting for potential confounding factors. Importantly, to the best of our knowledge, this is the first study to establish a significantly positive linear correlation between the TyG index and the incidence of visceral obesity. Therefore, our findings suggest that an elevated TyG index was associated with an increased risk of visceral obesity in patients with GC.

The radical gastrectomy remains the primary and most effective approach for the treatment of GC. However, the incidence of postoperative complications following radical gastrectomy ranges from approximately 12.5–51.0%, which demonstrates a significant association with tumor recurrence and poor survival outcomes (18). Visceral obesity has been recognized as a substantial risk factor for postoperative complications following gastrectomy in patients with GC (4, 19). On the one hand, this can be attributed to the excessive accumulation of visceral fat tissue that impedes the accurate identification of organs, vessels and lymph nodes, potentially resulting in prolonged operative duration, increased intraoperative blood loss and heightened surgical complexity. On the other hand, visceral fat exhibits a robust association with IR and adipocytokine-mediated inflammation, potentially compromising the normal response to surgical stress and elevating the susceptibility to postoperative complications (19).

Interestingly, Mao et al. found that compare to open gastrectomy, laparoscopic gastrectomy significantly mitigated the incidence of postoperative complications in patients with visceral obesity due to its advantages in terms of visual field and operating space (20). Additionally, they also conducted a comparative analysis on the incidence of postoperative complications among GC patients with visceral obesity who underwent different reconstruction methods. The results revealed that Billroth-I (B-I) reconstruction effectively mitigated the occurrence of postoperative complications and facilitated postoperative recovery (21). Moreover, based on a recent systematic review and meta-analysis, robot-assisted gastric cancer surgery in patients with visceral obesity demonstrates a significant association with reduced incidence of major complications compared to laparoscopic surgery (22). Therefore, accurate identification of patients with visceral obesity prior to surgery is crucial for selecting appropriate surgical procedures and reconstruction methods, thereby reducing the incidence of postoperative complications in GC patients.

Emerging evidence suggests a significant association between visceral obesity and IR. The TyG index, a validated and cost-effective indicator of IR, has been confirmed as a valuable predictor for diverse medical conditions (23), specifically those linked to insulin resistance and metabolic diseases such as metabolic syndrome (24, 25), nonalcoholic fatty liver disease (NAFLD) (26, 27), and type 2 diabetes mellitus (T2DM) (28, 29). Considering the strong correlation between the excessive accumulation of visceral adipose tissue and metabolic diseases, the TyG index may serve as a valuable predictor of visceral obesity. Yang et al. reported an independent association between the TyG index and visceral obesity in both males (odds ratio [OR] = 2.997) and females (OR = 2.233) among patients with T2DM (30). Consistent with these research findings, our study also demonstrated a dose-dependent positive correlation between elevated TyG levels and an increased risk of visceral obesity in patients with GC. However, the subgroup analysis revealed the TyG index was not associated with visceral obesity in GC patients with diabetes in our study. It is noteworthy that our study included a limited cohort of diabetic patients, thus necessitating larger sample sizes for further validation. Furthermore, a diabetic patient on hypoglycemic drugs with well-achieved glycemic control may exhibit a falsely low TyG index, yet they could still have visceral obesity. Consequently, the association between the TyG index and the risk of visceral obesity in diabetic patients was not evident in this study. A previous study has demonstrated a significantly stronger association between TyG and the risk of NAFLD in non-obese individuals compared to obese individuals, suggesting that the predictive efficacy of TyG for NAFLD risk is partially influenced by individual BMI (26). In our study, when conducting subgroup analysis stratified by BMI, we also observed a significantly stronger association between TyG and the risk of visceral obesity in patients with higher BMI than those with lower BMI. One possible explanation for this observation may be the higher prevalence of visceral obesity and elevated TyG levels among obese patients. This finding suggests that BMI plays a pivotal role in modulating the effectiveness of TyG as a marker for identifying patients at risk of visceral obesity.

Multiple lines of evidence consistently indicate a distinct sex disparity in the distribution of adipose tissue, with females exhibiting a higher proportion of subcutaneous fat, while males tend to accumulate a greater amount of visceral fat (31, 32). This can be partly attributed to the influence of sex hormones and their receptors (32). Consistent with previous findings, our study also observed a significantly higher level of visceral fat in males and a predominance of subcutaneous fat deposition in females. Additionally, our results demonstrated that being male was an independent risk factor associated with visceral obesity (Figure 4). Therefore, the sex disparity in adipose tissue should be taken into account when investigating an individual’s susceptibility to visceral obesity.

The present study had several strengths and limitations. The strengths of this study encompassed the utilization of more robust CT-based assessments for quantifying fat mass, adjustment for potential confounding factors such as demographic parameters and laboratory assays to minimize residual bias, consideration of target independent variables as both continuous and categorical variables to reduce dependence on specific data analysis methods, and the inclusion of subgroup analyses. However, it is important to acknowledge certain limitations in this study. Firstly, the cross-sectional design of the study warrants caution in drawing definitive conclusions; therefore, further research is necessary to validate the findings of this study. Moreover, only patients with GC were included in this study. It is noted that malignant tumors are associated with catabolism and negative energy balance, leading to changes in visceral fat. Consequently, there remains uncertainty regarding the generalizability of these findings to healthy populations or individuals with other diseases. Furthermore, this study included 36 (11.46%) patients on hypoglycemic drugs and 9 (2.87%) patients on lipid-lowering drugs, which may potentially result in a falsely low TyG index. This may have influenced the robustness of our findings. Lastly, it should be noted that anthropometric measurements such as waist circumference were not assessed in this study, which could potentially act as confounding variables when evaluating visceral obesity.

In conclusion, the TyG index demonstrated a significant association with visceral obesity and proved to be a valuable predictor for assessing visceral obesity when combined with BMI in patients with GC. Moreover, the TyG index can be easily obtained through laboratory tests in clinical settings. Therefore, the TyG index could serve as a straightforward and effective tool for evaluating visceral obesity.

## Data Availability

The raw data supporting the conclusions of this article will be made available by the authors, without undue reservation.
