# Supplementary material for: Triglyceride-glucose index as a marker for visceral obesity in patients with gastric cancer
Source: Front Nutr. 2025 Jan 10;11:1515918. doi: 10.3389/fnut.2024.1515918 (PMC11757132; doi:10.3389/fnut.2024.1515918)
Supplement: Supplementary file 1 [file Supplementary_file_1.docx]

**Table S1.** Univariate analyses of risk factors associated with visceral obesity

|  | OR | 95% CI | *P*-value |
| --- | --- | --- | --- |
| Age, years | 0.10 | 0.97-1.02 | 0.850 |
| Sex, n (%) |  |  |  |
| Female | Reference |  |  |
| Male | 2.35 | 1.42-3.88 | <0.001 |
| BMI, kg/m^2^ | 1.65 | 1.46-1.86 | <0.001 |
| Hypertension, n (%) | 1.86 | 1.19-2.91 | 0.007 |
| Diabetes, n (%) | 1.78 | 0.92-3.45 | 0.089 |
| ECOG performance status, n (%) |  |  |  |
| 0 | Reference |  |  |
| 1 | 0.82 | 0.49-1.37 | 0.451 |
| ≥2 | 0.32 | 0.15-0.66 | 0.002 |
| TNM stage, n (%) |  |  |  |
| Ⅰ | Reference | Reference |  |
| Ⅱ | 0.71 | 0.36-1.41 | 0.330 |
| Ⅲ | 0.48 | 0.27- 0.84 | 0.011 |
| Ⅳ | 0.50 | 0.23-1.07 | 0.074 |
| Hypoproteinemia, n (%) | 0.71 | 0.44-1.16 | 0.170 |
| Anemia, n (%) | 0.51 | 0.32-0.80 | 0.004 |
| [CRP](javascript:;)≥5mg/L, n (%) | 1.72 | 0.99-2.99 | 0.053 |
| NLR | 1.01 | 0.89-1.15 | 0.863 |
| LDL, mmol/L | 2.17 | 1.49-3.14 | <0.001 |
| HDL, mmol/L | 0.25 | 0.12-0.50 | <0.001 |
| TC, mmol/L | 1.38 | 1.08-1.77 | 0.011 |
| TyG index | 4.72 | 2.85-7.81 | <0.001 |
| TyG group |  |  |  |
| Low | Reference |  |  |
| Middle | 2.97 | 1.68-5.26 | <0.001 |
| High | 5.62 | 3.11-10.19 | <0.001 |

BMI, body mass index; CRP, [C-reactive protein](javascript:;); ECOG, Eastern cooperative oncology group; HDL, high-density lipoproteins; LDL, low-density lipoproteins; NLR, neutrophil-to-lymphocyte ratio; TC, total cholesterol; TNM, tumor–node–metastasis; TyG, triglyceride and glucose index.


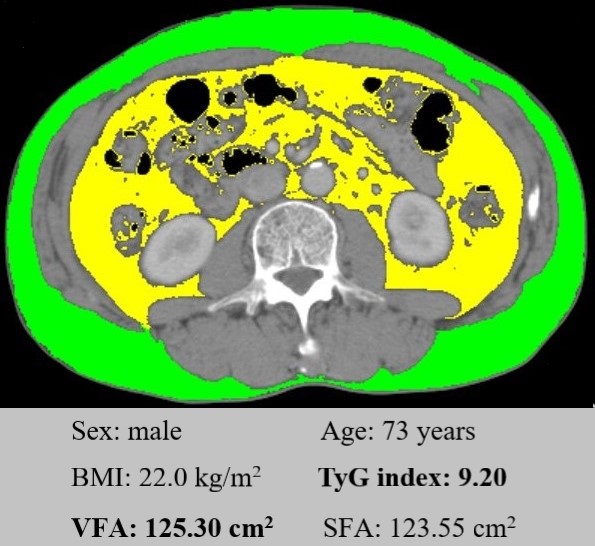


**Figure S1.** The analysis of CT images at L3 using the Slice-O-Matic software. Green indicates subcutaneous fat tissue, and yellow indicates visceral fat tissue. BMI, body mass index; L3, the level of the third lumbar vertebra; SFA, subcutaneous fat area; TyG, triglyceride and glucose index; VFA, visceral fat area.


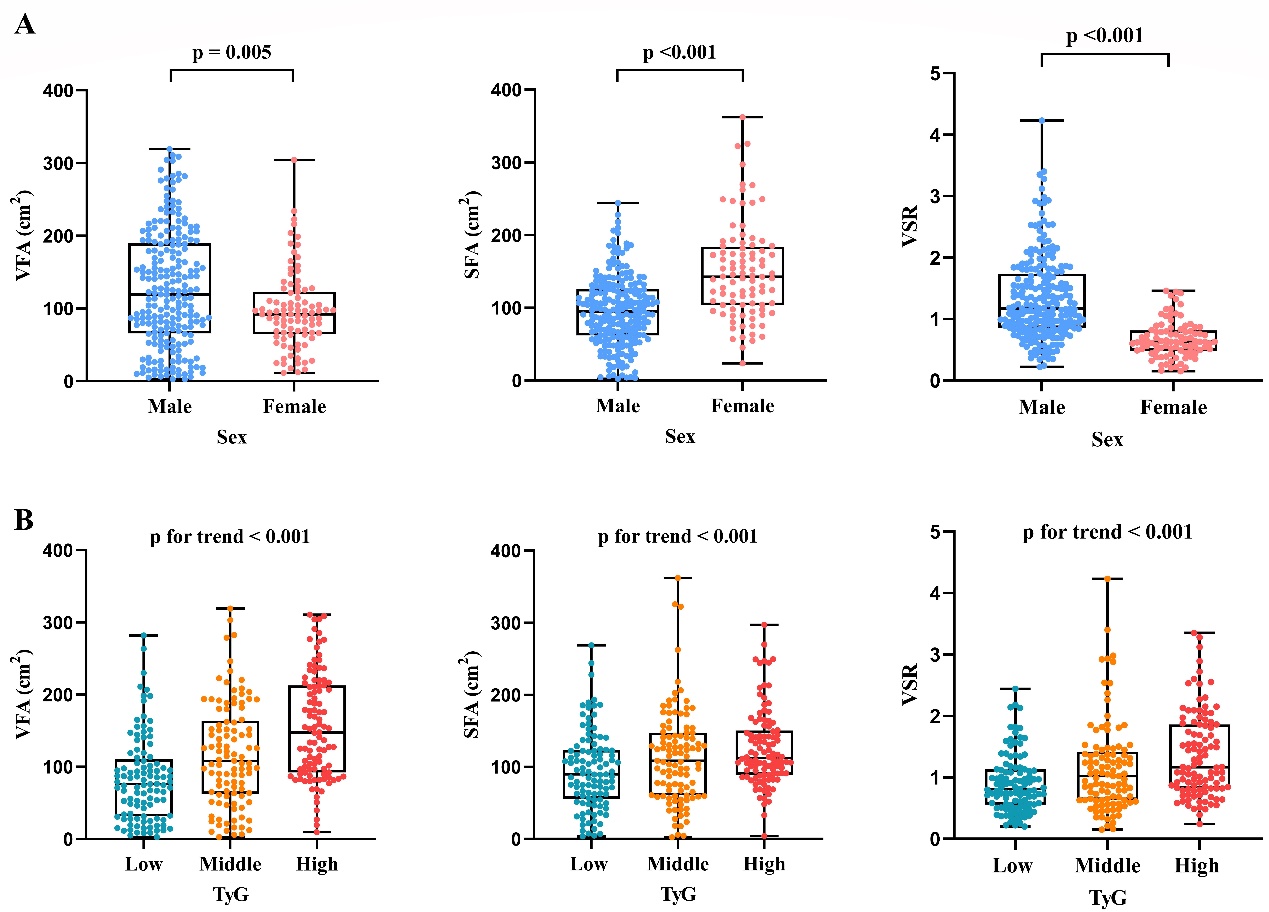
**Figure S2.** The comparison of VFA, SFA, and VSR levels based on (A) sex and (B) tertiles of the TyG index. SFA, subcutaneous fat area; TyG, triglyceride and glucose index; VFA, visceral fat area; VSR, VFA-to-SFA ratio.
